# Anopheline and human drivers of malaria risk in northern coastal, Ecuador: a pilot study

**DOI:** 10.1186/s12936-020-03426-y

**Published:** 2020-10-02

**Authors:** James A. Martin, Allison L. Hendershot, Iván Alejandro Saá Portilla, Daniel J. English, Madeline Woodruff, Claudia A. Vera-Arias, Bibiana E. Salazar-Costa, Juan José Bustillos, Fabián E. Saénz, Sofía Ocaña-Mayorga, Cristian Koepfli, Neil F. Lobo

**Affiliations:** 1 Eck Institute for Global Health, University of Notre Dame, Notre Dame, IN 46556 USA; 2 Centro de Investigación Para La Salud en América Latina, Facultad de Ciencias Exactas Y Naturales, Pontificia Universidad Católica del Ecuador, Calle San Pedro Y Pambahacienda, 170530 Nayón, Ecuador

**Keywords:** *Nyssorhynchus albimanus*, *Anopheles calderoni*, *Plasmodium falciparum*, Bionomics, Malaria, Long-lasting insecticide-treated nets (LLINs), Indoor residual spraying (IRS), Human behaviour, Ecuador

## Abstract

**Background:**

Understanding local anopheline vector species and their bionomic traits, as well as related human factors, can help combat gaps in protection.

**Methods:**

In San José de Chamanga, Esmeraldas, at the Ecuadorian Pacific coast, anopheline mosquitoes were sampled by both human landing collections (HLCs) and indoor-resting aspirations (IAs) and identified using both morphological and molecular methods. Human behaviour observations (HBOs) (including temporal location and bed net use) were documented during HLCs as well as through community surveys to determine exposure to mosquito bites. A cross-sectional evaluation of *Plasmodium falciparum* and *Plasmodium vivax* infections was conducted alongside a malaria questionnaire.

**Results:**

Among 222 anopheline specimens captured, based on molecular analysis, 218 were *Nyssorhynchus albimanus*, 3 *Anopheles calderoni* (n = 3), and one remains unidentified. Anopheline mean human-biting rate (HBR) outdoors was (13.69), and indoors (3.38) (*p* = 0.006). No anophelines were documented resting on walls during IAs. HBO-adjusted human landing rates suggested that the highest risk of being bitten was outdoors between 18.00 and 20.00 h. Human behaviour-adjusted biting rates suggest that overall, long-lasting insecticidal bed nets (LLINs) only protected against 13.2% of exposure to bites, with 86.8% of exposure during the night spent outside of bed net protection. The malaria survey found 2/398 individuals positive for asymptomatic *P. falciparum* infections. The questionnaire reported high (73.4%) bed net use, with low knowledge of malaria.

**Conclusion:**

The exophagic feeding of anopheline vectors in San Jose de Chamanga, when analysed in conjunction with human behaviour, indicates a clear gap in protection even with high LLIN coverage. The lack of indoor-resting anophelines suggests that indoor residual spraying (IRS) may have limited effect. The presence of asymptomatic infections implies the presence of a human reservoir that may maintain transmission.

## Background

While in the past 20 years there has been considerable success in terms of the reduction of malaria cases worldwide, there have been recent stalls in the outcomes of these efforts [1]. Between 2016 and 2017 there was a worldwide increase of reported malaria cases by approximately 2 million. Despite this, there were 20 million fewer reported malaria cases in 2017 when compared to 2010 [1]. Considering that malaria eradication is re-established as a goal on the global health agenda, it is critically important to understand why the previous Global Malaria Eradication Programme (GMEP) did not achieve its goals, thereby ensuring that present efforts are effective [2]. In order to achieve malaria elimination, programmes need to approach malaria transmission from multiple perspectives, including effective control of the parasite reservoir in both vector and human hosts.

Effective control and elimination efforts resulted in Ecuador having a considerable decrease in malaria cases since 2001. In 2012, Ecuador moved into pre-elimination phase [3]. The World Health Organization (WHO) included Ecuador in the E-2020 Initiative, together with other countries that were identified as being likely to reach zero indigenous cases by 2020 [1]. However, Ecuador has since seen a resurgence of malaria cases, both indigenous and imported [4, 5]. The highest number of cases since 2010 were reported in 2019 (n = 2081), with the lowest number of cases being reported in 2014 (n = 242) [1, 6]. Focal malaria transmission persists throughout the country, particularly in the northwestern coastal province of Esmeraldas as well as the Ecuadorian Amazon [6]. Moreover, the malaria elimination efforts are complicated by the presence of asymptomatic and imported cases [5, 7].

Currently, WHO-recommended interventions implemented in Ecuador include focally distributed long-lasting insecticide treated nets [LLINs], and indoor residual spraying (IRS), along with diagnosis and treatment with artemisinin-based combination therapy (ACT) [1, 8]. Ecuador has also implemented entomological surveillance to support elimination efforts [9].

While both IRS and LLINs have demonstrated to be effective in many settings against endophilic and endophagic malaria vectors [10, 11], regional effectiveness would depend on the bionomic traits of local vectors. An important indicator of LLIN effectiveness is human behaviour, a significant factor for personal protection. The risk of infectious bites increases when peak-biting behaviour occurs both outside of sleeping times and in spaces without LLINs [12, 13]. Routine household activities occurring in the morning and early evening, outside LLIN functionality, can result in gaps in protection that limit intervention effectiveness [14–16].

Data generated in Southern Ecuador indicate variation in seasonal vector species composition and temporal biting behaviour [17]. *Nyssorhynchus albimanus* (formerly *Anopheles albimanus*) demonstrated exophilic and exophagic behaviour with variation in these behaviours based on geography [17]. Data on vector species composition, human biting rate (HBR) and intervention-relevant behaviour of vectors from other endemic areas remain limited. Reports indicate that selective pressure exerted by indoor intervention strategies may result in mosquito behaviour shifting outdoors and/or later in the morning and early evenings, times and spaces with less intervention presence or use [11, 18, 19], pointing to the importance of a baseline understanding of transmission drivers and continued monitoring [20].

Historically, in coastal Ecuador, peak malaria transmission occurred in the first half of the year [21–23]. However a decrease in incidence, combined with focal outbreaks has resulted in this pattern being variable, with more cases being reported during November and December (2017, 2018–2019) as well as May and June (2017) [6, 24, 25]. Focal malaria transmission may have been exacerbated by increased rainfall and natural disasters that resulted in limited vector control resources, increased larval habitat availability, damaged infrastructure, and people spending more time outdoors [26, 27]. In addition, migration between neighbouring coastal communities, both in Colombia and Ecuador, risks introducing parasites into areas with decreased transmission, potentially causing outbreaks [28, 29].

The increasing number of malaria cases, a lack of knowledge of local entomological drivers of transmission, and with intervention strategies being implemented without understanding their protective efficacy may compromise the elimination agenda in Ecuador. Towards filling these gaps in knowledge in northwest coastal Ecuador, as well as understanding gaps in protection that result in continuing malaria transmission, this pilot study evaluated bionomic characteristics of anopheline species in relationship to human behaviour, human infections, and interventions present to inform on regional vector control strategies.

## Methods

### Study site

**Fig. 1:**
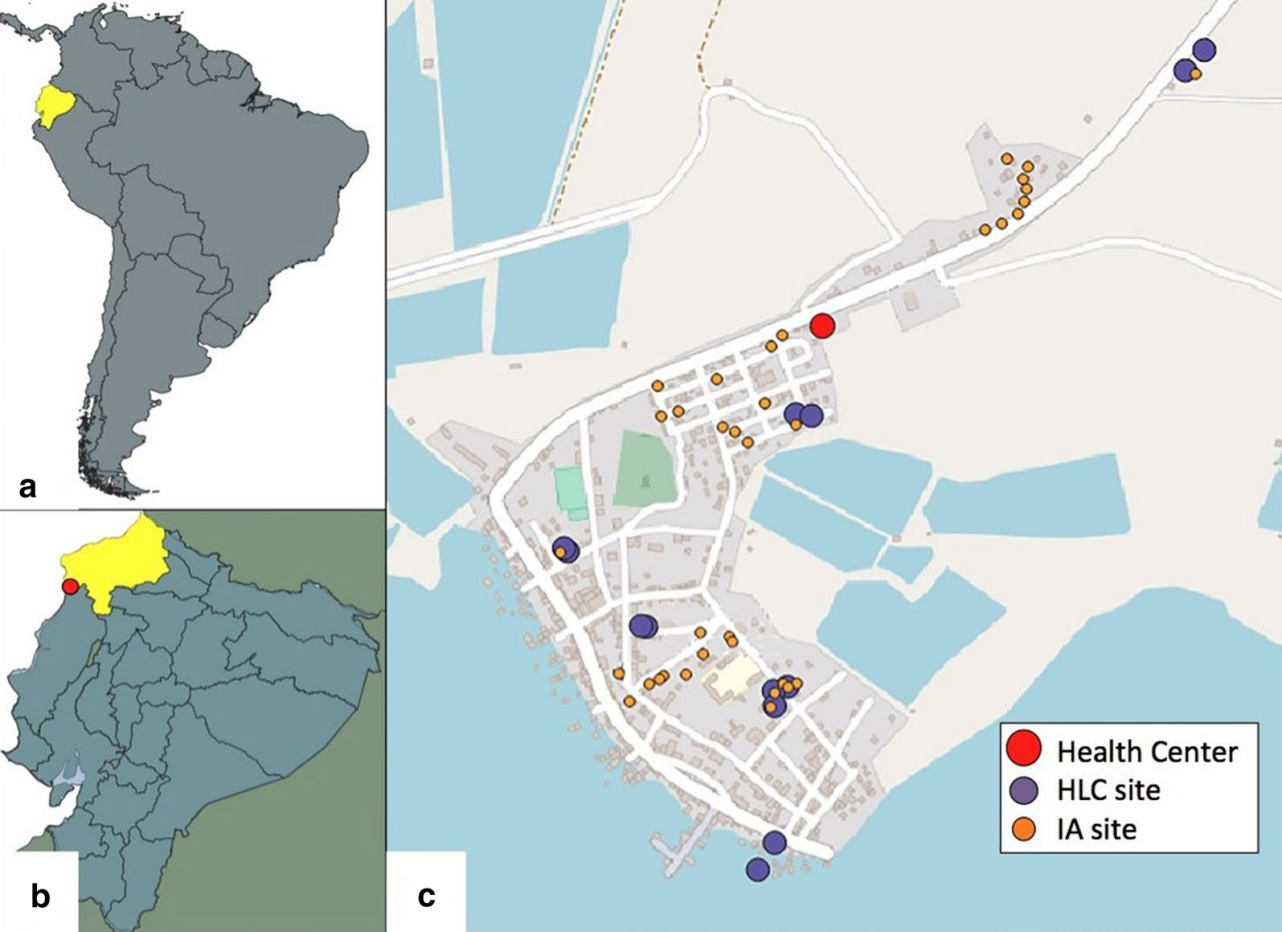
Map of the San José de Chamanga study site. **a** The location of Ecuador (yellow) in South America. **b** Location of Esmeraldas province (yellow) in Ecuador, with the study site marked in red. **c** The study site with the health centre (red), HLC (purple) and IA (orange) sites indicated

The study was conducted in San José de Chamanga (Fig. 1), a coastal community in Muisne county located in the southwest portion of Esmeraldas province, Ecuador (0° 16′ 10591.3" N 79° 57′ 16.973" W). The climate is tropical with an average temperature of 25.3 °C, the warmest month being April (average of 26.3 °C) and the coldest being September (average of 24.4 °C). Annual rainfall averages 1379 mm, with precipitation varying 242 mm between the driest month (November) and the wettest month (March). A major proportion (33.03%) of economic income is related to fishing, followed by tourism 4.87% and manufacturing 1.98%. Agriculture and livestock are mainly for subsistence and internal consumption [30].

According to the 2010 Census, all inhabitants in San José de Chamanga lacked basic needs, including access to drinking water, appropriate sewage treatment and garbage disposal [31]. Being part of an earthquake that occurred in April 2016, San José de Chamanga experienced significant damage to its infrastructure. Housing structure underwent radical changes with approximately 85% of pre-earthquake houses (generally with wood or cane walls and zinc roof and open eaves) being affected [32, 33]. Housing solutions, led by the Ministry of Urban Development and Housing (MIDUVI), included concrete-block houses as well as concrete and metal prefabricated houses. However, these houses usually do not present open eaves, high temperatures result in windows and doors being left open. Wood and cane houses with open eaves and open spaces in the walls are still present.

There were 146 reported cases of malaria in 2019 in the province of Esmeraldas [6]. The Vector-borne Diseases bulletin of the Ministry of Health indicated 28 *Plasmodium falciparum* infections in 2017 in San José de Chamanga (population 4,365), and 4 in 2018 [24, 25]. No malaria cases in Muisne county were reported during 2019 and 2020 (until epidemiological week 19) [6, 34].

### Adult mosquito collections

Human landing catches (HLCs) [35], performed by both researchers and trained local adults, were conducted to sample human host-seeking mosquitoes. Researchers accompanied local collectors in problem solving and supervision. Informed consent to participate in mosquito sampling was received from local inhabitants after the objectives, activities, risk and benefits were explained. A rapid test for malaria diagnosis was conducted for all mosquito collectors at the health centre prior to the first and two weeks after the last mosquito collection date towards treating any diagnosed malaria case. Treatment was available, if needed, free of charge as part of the Ministry of Health programme. A positive diagnosis also necessitated exclusion from HLC activity. Collections took place for six nights within a period of two weeks (three consecutive nights per week) in 13 different houses (Fig. 1), in May 2019. Each HLC house had one indoor and one outdoor HLC collector throughout the duration of each collection period.

Collections conducted during the first week took place between 18.00 and 06.00 h. The first week of collections indicated the possibility of mosquitoes host seeking earlier than the 18.00 start time. To enable the capture of this possible earlier host seeking, HLC collections during the second week started at 16.00 (2 h earlier) and lasted until 06.00. All nightly collections took place for 50 min each hour with 10 min of rest. Mosquitoes were held in individual waxed paper cups, labelled with the hour of collection, location (indoor or outdoor) and a unique household code. Mosquitoes were killed by ethyl acetate or by freezing, morphologically identified to genus, and placed in individually labelled 1.5-ml eppendorf tubes with silica gel.

### Indoor resting aspiration (IAs)

A total of 36 houses, distinct from HLC houses, were examined for resting mosquitoes. Indoor resting aspiration (IA) houses were selected to include different types of local structure (block houses, prefabricated houses, concrete and metal, and wood/cane houses). All houses of the community were within a 1-km radius of the health centre, and AI houses were spatially distributed within this space (Fig. 1). Between 06.00 and 08.00 an investigator entered each house and inspected the walls of each bedroom for resting mosquitoes. Resting anophelines were collected with a mouth aspirator and stored in individually labelled Eppendorf tubes as above.

### Species identification

Each anopheline specimen was morphologically identified to species, using a taxonomic key [36]. For molecular confirmation of species, genomic DNA was extracted from the whole body specimens using a sodium hydroxide extraction method [37]. Molecular identification was determined by sequencing the internal transcribed spacer region 2 (ITS2) and the cytochrome oxidase subunit 1 (CO1) loci. The ribosomal ITS2 [38–40] and mitochondrial CO1 [41] loci were amplified by PCR, and Sanger sequenced on ABI 3730xl DNA analyzer platform (PE Applied Biosystems). ITS2 and CO1 sequences were aligned with a minimum match percentage of 98% and 95%, respectively, using Seqman Pro assembly software (Lasergene v 12.3.1). Contiguous sequence assemblies were trimmed and examined manually for quality, any contaminated and poor-quality sequences were removed from the analysis. Nucleotide BLASTs (NCBI) of sequence assemblies was used for final species determination.

### Human behaviour

Human behaviour observation data (HBOs) were collected in HLC houses alongside HLC collections. The number of humans (both household and non-household members) present both within the house and outdoors (within 10 m of the house) were recorded at the beginning of each HLC collection hour by the HLC collector for that hour and location of collection. The HLC collector was excluded from HBO count data. LLIN usage as well as the time people went to sleep were also noted. Data were recorded on datasheets by the HLC collector, checked by a researcher, and entered into an Excel spreadsheet.

### Analysis

The human biting rate (HBR) was calculated as the mean number of mosquitoes biting per person per location (indoors/outdoors) per time period (hour or night). Bionomic data were analysed statistically using SPSS version 26 (IBM Corp., Somers, NY, USA). Degree of normality among the data was determined using a Shapiro–Wilk test. HBR means were compared using non-parametric Wilcoxon Sign Rank tests based on Shapiro–Wilk test results. Human behaviour observation-adjusted HBRs (HBO-adjusted HBRs) were calculated based on Monroe, [15]. Here, directly observed mosquito biting rates were used alongside the HBO observations and data from the cross-sectional survey [14, 15, 42]. The percentage of the study population asleep during each hour of HBO observation was considered in analyses. LLIN use (people asleep under LLINs) determined in the survey was used for analysis.

### Cross-sectional survey

Venous blood samples were collected from 398 individuals of ages 2 and older spread across the Chamanga community and screened for *P. falciparum* and *Plasmodium vivax* by rapid diagnostic test (RDT, AccessBio CareStart PfHRP2/PvLDH Combo Test). DNA was extracted from 100 µL blood using the Macherey–Nagel NucleoMag kit. Four µL of DNA (corresponding to 4 µL of blood) was screened for *P. falciparum* using the varATS assay [43] and for *P. vivax* using the *cox1* assay [44]. A brief questionnaire (Additional file 1) including LLIN use, recent symptoms of febrile illness, and recent travel were also conducted with the head of the household, concurrent to the blood sample collections and after informed consent.

### Ethical considerations

The study obtained ethical clearance from the Institutional Review Boards of the University of Notre Dame, Notre Dame, Indiana, USA, the *Pontificia Univesidad Católica del Ecuador*, and the Ministry of Health of Ecuador. Mosquitoes were collected under the Ecuadorian agreement MAE-DNB-CM-2015-0030-M-0001. Community members, in the presence of health personnel, were informed about the objectives, procedures, potential risks and benefits related to the study. Informed consent was obtained from local HLC collectors, the heads of households where IAs and HLCs were performed, and the participants of the blood survey and questionnaire.

## Results

### Vector species composition

Anopheline mosquitoes (n = 222) were collected over 13 nights using HLCs. Of these, 208 (93.7%) were morphologically identified as *Nyssorhynchus albimanus*, 10 (4.5%) as *Anopheles calderoni*, and 4 (1.8%) could not be identified to species (*Anopheles *spp.). Molecular identification using ITS2 and/or CO1 sequences confirmed species identities for 198 specimens. Three of the 4 unidentified specimens were identified as *Ny. albimanus.* Six specimens of *Ny. albimanus* were morphologically misidentified *An. calderoni*, while a single *An. calderoni* specimen was misidentified as *Ny. albimanus*. Molecular analysis of the remaining samples (n = 24) did not work with either ITS2 or CO1 after multiple attempts at amplification, attributed to sample degradation. Final species descriptions were based on morphology, as well as molecular identification (Table 1). Analysis of behaviours was conducted on *Ny. albimanus* alone, while exposure-based analyses were conducted on all anopheline specimens.

**Table 1:** The number of specimens and proportion of each species collected by HLCs—indoors and outdoors, in San José de Chamanga, Ecuador

Species (based on molecular and morphological results)	Indoor n (%)	Outdoor n (%)	Indoor HBR	Outdoor HBR	Total n (%)
*Ny. albimanus*	43 (19.8)	175 (80.2)	3.30	13.46	218 (98.2)
*An. calderoni*	1 (33.3)	2 (66.6)	–	–	3 (1.3)
*An. *spp. (not identified)	0 (0.0)	1 (100.0)	–	–	1 (0.4)
Total (all anophelines)	44 (19.8)	178 (80.2)	3.38	13.69	222 (100)

The indoor and outdoor biting rates (bites per person per night) are shown for the major species (*Ny. albimanus*) and all anophelines

### Vector bionomics: host-seeking behaviour

**Fig. 2:**
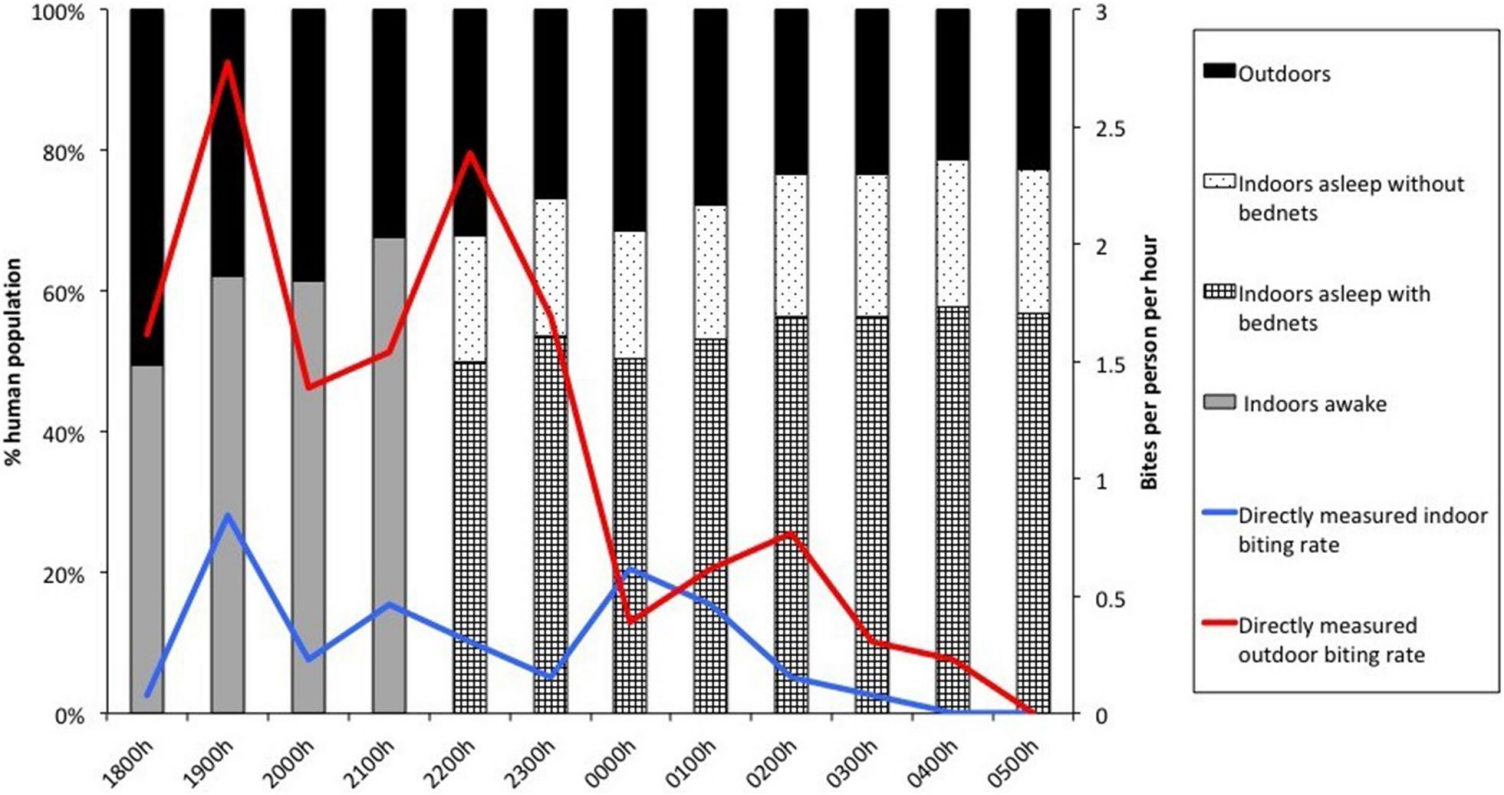
Anopheline and human behaviours. Directly observed anopheline biting rates (based on HLCs) are outlined in red (outside) and blue (inside) throughout the night. The proportions of humans in 4 behavioural groups: outdoors, indoors awake, indoors asleep with LLINs, and indoors asleep without LLINs, are depicted in the bar graph. People went to sleep at 22.00 while 73.4% of inhabitants used bed nets

Directly observed host seeking, as characterized by HLCs, was documented throughout the night with an indoor peak between 19.00 and 20.00 and outdoor peaks between 19.00 and 23.00 (Fig. 2). These peaks were followed by general decline in landing rates during the rest of the night. No anophelines were captured between 16.00 and 18.00, and these hours were excluded from analyses.

*Nyssorhynchus albimanus*, the principal vector collected was primarily collected outdoors (80.2%) with an indoor HBR of 3.3 and an outdoor biting rate of 13.46 bites per person per night*.* The overall outdoor anopheline HBR was approximately four times higher than the indoor HBR: 13.69 *versus* 3.38, *p* = 0.006, Table 1).

### Vector bionomics: indoor resting behaviour

Morning IAs were conducted towards determining indoor resting densities of anopheline mosquitoes. No anophelines were seen or captured resting on walls within any of the 36 houses selected for IAs. *Culex* mosquitoes were observed resting on house walls, LLINs, and on other surfaces within IA houses.

### Human behaviour observations

Human behaviours related to location (inside or outside), sleep, and LLIN use (people asleep under LLINs) were characterized alongside HLCs, and through a questionnaire (Fig. 2). HLC participants in each HLC house documented the number of people inside and outside the house and the hour at which residents went to sleep. People outdoors increased between 18.00 and 19.00 and subsequently decreased as people moved indoors to sleep. A proportion of people remained outside throughout the night without interventions being used. HLC participants observed the times at which community members turned house lights off (to go to sleep). Overall, this was at 22.00 with people awakening at 05.00. Ten of the 13 HLC houses had at least one LLIN in use, while all 36 IA houses had LLINs in use. These observations were corroborated by data collected from 79 houses in the concurrent cross-sectional survey (Additional file 1), where 73.4% (n = 58) survey respondents stated that they slept under a LLIN the previous night. The 73.4% LLIN use documented in the survey was used in analyses.

### Human behaviour-adjusted vector biting rates

**Fig. 3:**
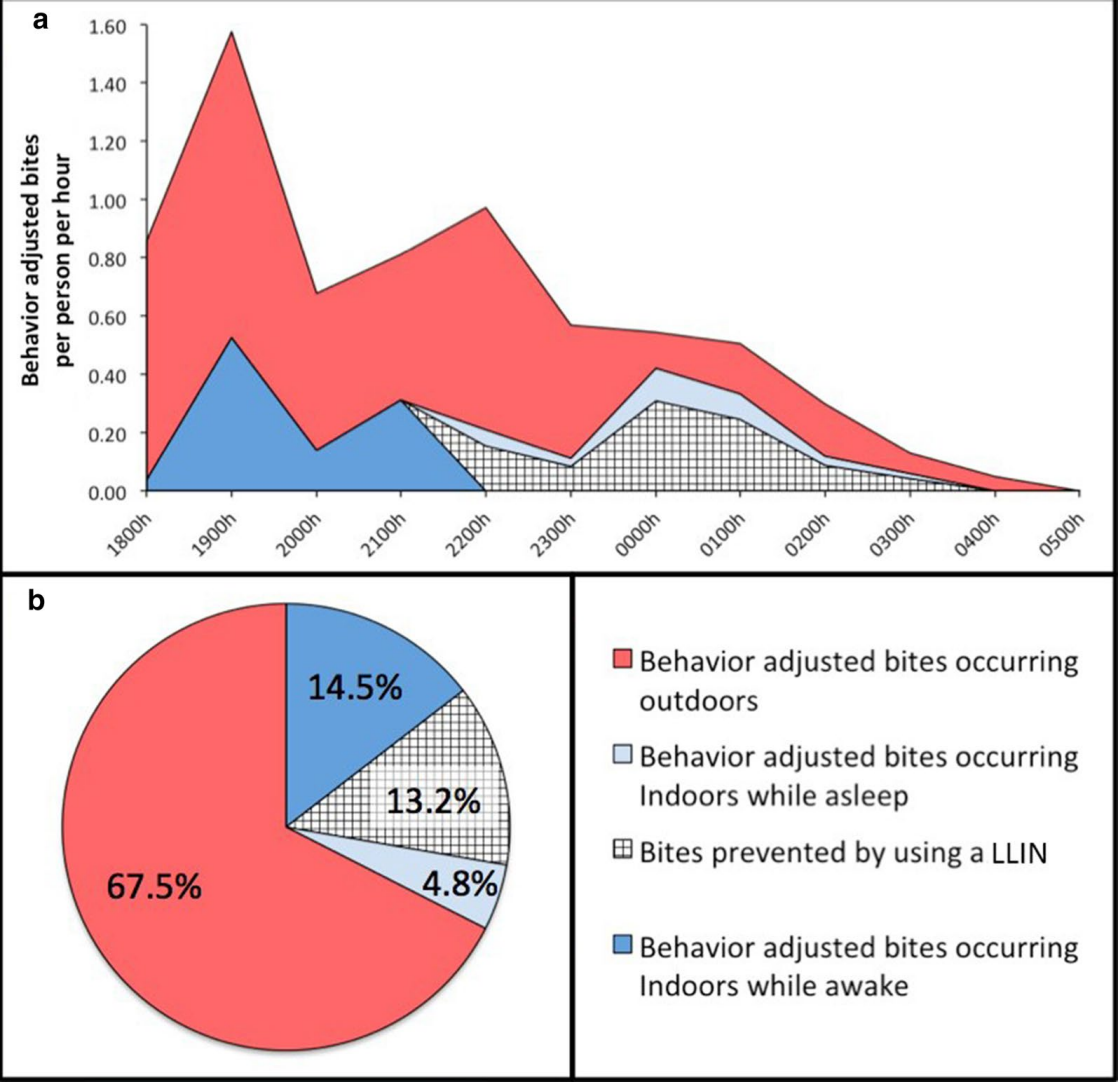
Human behaviour-adjusted biting rates. **a** Adjusted biting rates are depicted over the course of the night based on the proportions of people inside and outside HLC houses, as well as time they went to sleep and bed net use. Bites prevented by bed net use are also depicted. **b** The proportion of biting in each group is outlined along with bites prevented by bed nets. Most biting occurs outside and early in the evening

Directly observed vector biting rates were adjusted to factor in human presence (inside or outside), time inhabitants went to sleep (22.00), and LLIN usage (73.4%, determined in the survey) (Fig. 3) [14, 15]. The adjusted analysis indicates that most biting occurs early in the evening and outside (67.5%), with 14.5% of biting occurring inside the house while people are awake and 4.8% of biting occurring inside the house while people are asleep and not using LLINs (Fig. 3). The present 73.4% LLIN usage prevents approximately 13.2% of biting. With the high amount of early evening biting, 10.7 and 19.3% of bites still occur indoors for users of LLINs and non-LLIN users, respectively.

### Malaria prevalence survey

#### Population prevalence of Plasmodium falciparum and Plasmodium vivax

By qPCR, 2/398 individuals were positive for *P. falciparum* at densities < 1 parasite/µl blood. Both individuals tested negative by rapid diagnostic test (RDT). No individuals tested positive for *P. vivax*. Individuals testing positive for *P. falciparum* included a 45-years-old housewife and a 25-years-old female industrial worker. Both positive individuals did not present symptoms and did not live in any of the houses in which entomology collections were performed. However, they did report that they each travelled outside of San Jose de Chamanga in the previous 2 weeks, in both cases to areas without malaria transmission: Portoviejo and Santo Domingo. At the time of blood collection, 32% of people reported symptoms of clinical illness (Symptoms Survey, Additional file 1). Of those with symptoms, the most common was headache (43%) and most people had symptoms for more than a week (42%). Only 23.4% of the people giving blood for the study reported having had malaria previously, out of which 62.5% reported only one episode of the disease.

### Questionnaire results

Community houses (n = 86) were surveyed for specific aspects of malaria (Questionnaire, Additional file 1). Sixty per cent of interviewed people were women, of whom 42% categorized themselves as housewives. The majority of respondents (62%) considered themselves as *mestizos* (of mixed descent) followed by afro-Ecuadorians (32%). Most respondents had either completed high school (43%) or left high school before its completion (40%). Seventy-four per cent reported that no one in their house had been diagnosed with malaria in the last 12 months. Most houses were made of brick (43%) or wood (39%), had electricity (76%) and had a toilet inside the house (64%). For half (50%) of the respondents, the main water source was fewer than 20 m from the house, and also used tankers (water trucks) as a source of water (72%). While most people (63%) knew that mosquitoes transmitted malaria, 37% did not. Most people (64%) knew that fever and headache (53%) are primary malaria symptoms. In general, there was a lack of malaria-related knowledge, more than half of respondents did not know how malaria should be treated or prevented. Approximately 28% of respondents believed that the use of long-sleeved shirts and pants could prevent malaria. The vast majority of respondents said that they sleep under a bed net (86%) with 73.4% reporting that they slept under a bed net the previous night. Of those interviewed, 22% consider spatial spraying as the main method to prevent malaria outside the house. The majority (44%) of respondents believed that the responsibility of malaria control lay with health workers, *versus* 24% saying that the community itself should be responsible. Even though 60% knew that mosquitoes are vectors of malaria, 57% believed that malaria is transmitted through physical contact. Major responses for all questions are reported in Additional file 1.

## Discussion

Malaria transmission is driven by the interactions between behaviour and vectorial capacity of local anopheline mosquitoes, human behaviour, and the interventions in use. A multi-pronged approach was utilized in an operational research pilot study towards understanding gaps in protection and reasons for continued malaria transmission in San José de Chamanga, Esmeraldas, on the Ecuadorian Pacific coast.

The primary outcome of this study documented that most exposure to the anopheline vectors occurs outdoors, and that the primary interventions used, LLINs and IRS, though useful, may have limited efficacy in further reducing malaria. LLINs function by killing exposed susceptible mosquitoes, physically protecting people from infectious bites, as well as providing community protection [45–47], while IRS functions by killing susceptible mosquitoes that rest on sprayed walls [48, 49]. These protections may be limited by vector behaviour that circumvent the intervention: outdoor or early biting behaviour, times and spaces where LLINs are not usually in use, or by a lack of indoor-resting behaviour. In addition, human usage of LLINs, as well if their presence overlaps biting anophelines in time and space, impacts intervention efficacy [14, 15]. Since outdoor human activities expose people to mosquito bites, social and cultural factors need to be taken into account when evaluating exposure [50]. In San José de Chamanga, the combination of vectors biting primarily outdoors and early in the evening, and inhabitants present outdoors without protection contributes to more than 65% of exposure to mosquito bites occurring outdoors. In addition, a hypothetical increase in LLIN use in all people asleep indoors, will only increase protection from the present 13.2 to 18.0%, indicative of highly exophagic vectors and outdoor human behaviours. Indoor aspirations did not catch any anophelines, pointing to the lack of overlap between how IRS functions and how local mosquitoes behave. The absence of indoor-resting anophelines in the morning (note that *Culex* were observed resting inside most IAs houses), however, does not preclude the possibility that anophelines entered households to feed, rested on indoor surfaces, and moved outdoors before IAs were conducted, indicating a possible impact of IRS. The presence of susceptible anophelines biting indoors still indicates an overall impact on the vector population, with those entering being impacted by exposure to LLINs [2, 10, 18]. The additional evaluation of insecticide resistance may impact the understanding of intervention efficacy. Human behavioural observations were also based on a limited number of households and population level variation in behaviours was not incorporated. The community-wide, self-reported LLIN use (73.4% utilized for analysis) was supported by household HBO data, where 76.9% (10 of 13 households) documented at least one LLIN in use. The use of HBOs from the same households from which HLCs were conducted enables a 1:1 evaluation of exposure to anopheline bites. In addition, the analysis of behaviour during time spent away from the household would enable an evaluation of possible exposure to bites outside the domestic and peri-domestic area.

These data emphasize the need for new tools, such as spatial [51] or topical repellents, as well as new research that evaluates novel paradigms directed at residual transmission occurring outside the functional scope of present interventions, accelerating progress towards elimination.

The northern Pacific coast in Ecuador has limited information on vector species and their behaviours. For the first time, anopheline species composition and biting behaviour were quantified and described for San José de Chamanga at the end of the rainy season. *Nyssorhynchus albimanus* was found to be the most abundant anopheline species collected in HLCs, followed by smaller numbers of *An. calderoni*. *Nyssorhynchus albimanus*, a primary vector of malaria in Central and South America (including Ecuador) [17, 36, 52–57], has location-specific bionomic traits [58]. Similar to behaviours seen here, *Ny. albimanus* has been documented as being exophagic (outdoor feeding) and exophilic (outdoor resting) [59], while in other countries, this species has demonstrated a preference for resting indoors [57]. Since behaviour of *Ny. albimanus* changes by geography, vector interventions that work against this species in one area may not work in another. Consequently, understanding local vector behaviour is important when evaluating both how a vector contributes to risk of exposure and also how interventions may function.

While collected in low numbers, this study presents the first confirmed report of *An. calderoni* in Esmeraldas province, Ecuador. Historically, *An. calderoni* has been misidentified as *Anopheles punctimacula*, *Anopheles malefactor* and *Anopheles guarao* due to their highly similar morphologies. However, the availability of ITS2 and CO1 sequencing allows for molecular identification that has improved the known distribution of this species in South America to include Ecuador, Peru and Colombia [52, 55]. This species has been documented as being a primary vector in southwest Colombia, with exophagic and early evening behaviour and higher populations coincident with rainy season, behaviour reflected in this study [53, 55]**.**

Only two asymptomatic cases of malaria were detected in the malaria survey (n = 398), a point prevalence of 0.5%. The low parasite density seen would not be detected by microscopy or RDTs; this combined with the lack of symptoms indicates that these cases may go undetected and untreated and possibly be long lived. Although malaria infections occurring below the limit of detection of standard diagnostics are present in all endemic settings, including Ecuador [7], their presence in areas with reduced malaria and those approaching elimination may be particularly important as they may be the primary contributor to the infectious reservoir, thereby sustaining transmission [60–62]. Although there was a history of travel in both malaria cases, the destination and duration of the travel indicate that the infections probably occurred in Chamanga or *en route*. These infections corroborate the need to maintain both entomological and epidemiological interventions to prevent outbreaks and further increases in disease incidence. Even without high levels of ongoing and endemic transmission, malaria may be regularly imported into the area, a risk factor considering the high receptivity and vulnerability of the population [63, 64], which may possibly lead to outbreaks, as in 2017, or further increases in transmission [65]. Serological analysis of blood samples from the survey may provide insights on recent malaria exposure in the community of Chamanga, and elaborate on local transmission. This would be useful in the determination of high-risk populations, and may inform targeted and focal intervention strategies based on exposure.

The proportion of people lacking knowledge of basic malaria transmission, prevention or treatment was considerably high when compared with other communities previously surveyed to the north of Esmeraldas province and on the Colombian Pacific coast [7, 66]. This lack of knowledge is attributed to declining malaria over the past decade in Chamanga, in contrast to communities in the north of Esmeraldas and the Pacific coast of Colombia where malaria is endemic. Education about malaria and other mosquito-transmitted diseases by health authorities is required in this region as outbreaks may still occur.

## Conclusions

The single time point captured in this study has increased our understanding of both gaps in protection as well as intervention effectiveness. More thorough evaluations across multiple transmission seasons could yield further data towards more effective vector control necessary for malaria elimination. The limited overlap between spatial and temporal vector-biting behaviours, human behaviours and interventions (LLIN and IRS) demonstrates that additional approaches are required to reduce continued residual malaria transmission and achieve elimination. The identification of asymptomatic *P. falciparum* carriers in the same population further corroborates the potential for outbreaks. This pilot study elucidated intervention functionality, identified gaps in protection, and pointed to where and how intervention strategies can be improved, focusing on comprehending interactions between entomological drivers, human behaviour and intervention-related factors.

## Supplementary information

**Additional file 1.** Demographic characteristics, symptoms and knowledge, attitude and practices (KAP) questionnaire among participants in San José de Chamanga, Esmeraldas, Ecuador

## Data Availability

Data supporting the conclusions and outcomes of this article are available upon request from the corresponding author.

## Abbreviations

HLC: Human landing catch
IA: Indoor resting aspiration
HBO: Human behavior observation
LLIN: Long-lasting insecticide net
IRS: Indoor residual spray
