# Supplementary material for: Anopheline and human drivers of malaria risk in northern coastal, Ecuador: a pilot study
Source: Malar J. 2020 Oct 2;19:354. doi: 10.1186/s12936-020-03426-y (PMC7532652; doi:10.1186/s12936-020-03426-y)
Supplement: Supplementary file 1 — Additional file 1. Demographic characteristics, symptoms and knowledge, attitude and practices (KAP) questionnaire among participants in San José de Chamanga, Esmeraldas, Ecuador [file 12936_2020_3426_MOESM1_ESM.docx]

**Additional file 1. Demographic characteristics, symptoms and knowledge, attitude and practices (KAP) questionnaire among participants in San José de Chamanga, Esmeraldas, Ecuador.**

**Symptoms survey (Conducted with all participants in the blood cross sectional survey):**

| **Interview question** | **Primary answer reported** | **Percentage** |
| --- | --- | --- |
| Interviewee sex | Women | 58.78 |
| Do you currently have symptoms of malaria? | No | 68.29 |
| If you do have symptoms, What are your current symptoms? | Headache | 42.74 |
| For how long have you had your present symptoms? | More than a week | 41.94 |
| Have you taken medication for your present symptoms? | No | 41.94 |
| If you did take medication, what type of medication did you take for your present symptoms? | Paracetamol | 29.84 |
| Have you ever had malaria? | No | 76.59 |
| How many times have you had malaria? | 1x | 62.50 |
|  | 2x | 15.63 |
| What is your profession? | Housewife | 43.90 |
| What is your maximum level of schooling? | Incomplete secondary school | 30.00 |

**Knowledge, attitude and practices survey**

| **Interview question** | **Primary answer reported** | **Percentage** |
| --- | --- | --- |
| Interviewee sex | Women | 60.23 |
| Has anyone in your household had malaria in the last 12 months? | No | 73.86 |
| How is malaria is transmitted? | Mosquito | 663.1 |
| What are malaria symptoms? | Fever | 63.64 |
| What is the first thing a person should do when they have symptoms of malaria? | Report to a microscopist or health officer for a thick blood smear test | 85.23 |
| Is a microscopist or health center In this community? | Yes | 48.86 |
| Does the microscopist or health center charge for testing? | Yes | 48.86 |
| What is the treatment for malaria? | Does not know | 52.27 |
| What happens if a person does not take malaria treatment? | Worsens and may die | 85.23 |
| What can be done inside the house to prevent malaria? | Wear long-sleeved shirt and pants | 27.27 |
| What can be done outside the home to prevent malaria? | Fumigate | 21.59 |
| Who is responsible for controlling malaria? | The health center | 44.32 |
| Who taught you what you know about malaria? | Health workers | 36.36 |
| Is getting sick from malaria common and normal? | No | 71.59 |
| Do pools or accumulated water around the house facilitate the transmission of malaria? | Yes | 88.64 |
| Is malaria transmitted through physical contact? | No | 56.82 |
| Do medications cure malaria? | Yes | 78.41 |
| Is using a bed net is annoying to you? | No | 65.91 |
| Does presence of mosquitoes bothers you? | Yes | 98.86 |
| Do you have to take a thick blood smear to know if you have malaria? | Yes | 84.09 |
| Should a person who is sick with malaria or malaria continue to take the prescribed medications even if they feel better | Yes | 95.45 |
| Have you bought over-the-counter pills to get relief from malaria? | No | 62.50 |
| Can you get malaria more than once? | Yes | 86.36 |
| What are the malaria prevention methods you use inside the house? | Mosquito nets and repellent spray | 43.18 |
| What are the malaria prevention methods you use outside the house? | Fumigate | 36.36 |
| When you or a family member as sick with malaria, who did you go to first? | Physician / Nurse / Nursing Assistant | 81.82 |
| When you or a family member went to a health center with malaria symptoms, did you get a thick blood smear? | Yes | 30.68 |
| Transportation: How did you travel to the place where you got a thick blood smear? | Foot | 68.18 |
| What do you think is the reason why people do not finish the malaria treatment? | They stopped it because they feel better | 64.77 |
| Is treatment for malaria free? | Yes | 77.27 |
| What is your occupation? | Housewife | 42.05 |
| What is your highest level of education? | Complete secondary school | 43.18 |
| What is your Ethnicity? | Mestizo | 61.36 |
| What public services do you have access to? | Electric energy | 76.14 |
| What is the primary type of housing you live in? | Brick material house | 43.18 |
| What is the distance is the nearest water source from your house? | Less than 20m | 50.00 |
| What is the primary source of water you use to prepare food | Water bottles and tanker | 71.59 |
| Where is your toilet located? | Inside the home | 63.64 |
| What type of toilet do you have? | Toilet connected to the septic tank | 61.36 |
| What type of lighting do you use? | Electric (Light bulbs) | 98.86 |
